# Co-Occurrence and Molecular Characterization of ESBL-Producing and Colistin-Resistant *Escherichia coli* Isolates from Retail Raw Meat

**DOI:** 10.3390/foods14203573

**Published:** 2025-10-21

**Authors:** Arife Ezgi Telli, Nihat Telli, Yusuf Biçer, Gamze Turkal, Tahir Yılmaz, Gürkan Uçar

**Affiliations:** 1 Food Hygiene and Technology Department, Veterinary Faculty, Selcuk University, Konya 42130, Türkiye; yusufbicer@selcuk.edu.tr (Y.B.); gamze.kabak@selcuk.edu.tr (G.T.); gucar@selcuk.edu.tr (G.U.); 2 Food Processing Department, Technical Sciences Vocational School, Konya Technical University, Konya 42250, Türkiye; ntelli@ktun.edu.tr; 3 HallMark Veterinary and Compliance Services, Gloucestershire GL13 9JR, UK; tahirylmz@icloud.com

**Keywords:** *E. coli*, ESBL, antibiotic resistance, colistin resistance, retail meat

## Abstract

**Background:** The emergence of extended-spectrum β-lactamase (ESBL) producing and colistin-resistant *Escherichia coli* in retail meat poses a significant public health risk. **Method:** A total of 180 retail meat samples (chicken parts, internals, processed products; lamb; beef; fish) were purchased from markets and butcher shops across Turkiye. Presumptive ESBL-producing isolates were screened on chromogenic agar and phenotypically confirmed. Species identity was verified by *uspA* PCR, and resistance genes (*blaCTX-M*, *blaTEM*, *blaOXA*, *blaSHV*, *mcr*-1, *mcr*-2, *mcr*-3) were analyzed. Colistin MICs were determined by broth microdilution, while antimicrobial susceptibility of ESBL-positive isolates was assessed by disk diffusion. **Results:** Overall, ESBL-producing *E. coli* were detected in 21.7% (*n* = 39) of the 180 meat samples analyzed, with the highest prevalence observed in chicken parts (26/40, 65.0%) and giblets (6/10, 60%). All ESBL-*E. coli* isolates harbored *blaCTX-M*, with *blaCTX-M-*1 identified as the sole variant. The *blaTEM* gene was detected in 61.5% (24/39) of ESBL-positive *E. coli* isolates. Colistin resistance was identified in six isolates (15.4%), all of which carried *the mcr-1 gene*. Additionally, one lamb minced meat isolate harbored *the mcr-2 gene*. Co-occurrence analysis revealed that the most frequent resistance gene combination among ESBL-producing isolates was *blaCTX-M1* + *blaTEM*, detected predominantly in chicken meat samples, while *mcr*-1 was observed only in isolates harboring single or limited resistance genes, suggesting a distinct acquisition pattern. **Conclusions:** A high prevalence of *blaCTX-M*-1 and the co-occurrence of *mcr* genes were detected in *E. coli* isolates from retail meat, particularly poultry. The detection of *mcr*-1/*mcr*-2 co-carriage in lamb meat, though rare, highlights the need for broader surveillance. These findings underscore the need for integrated monitoring and prudent antimicrobial use in food animals. The use of antibiotics as growth promoters is prohibited in Türkiye, and therapeutic applications require a veterinary prescription; however, stronger enforcement remains essential to limit the dissemination of multidrug-resistant bacteria in the food chain.

## 1. Introduction

Antibiotic use in animal production, whether for therapeutic, prophylactic, or growth-promoting purposes, has been a major driver of antimicrobial resistance, leading to the emergence of multidrug-resistant foodborne pathogens that can be transmitted to humans through the food chain or direct contact with animals. These resistant strains may contaminate different meat sources, with their persistence and dissemination shaped by processing and handling conditions. Of particular concern is the increasing prevalence of extended-spectrum β-lactamase (ESBL)-producing Enterobacterales and the emergence of plasmid-mediated colistin resistance genes (*mcr*), both of which compromise last-resort therapies in clinical practice and represent a serious global health threat [1]. Despite growing international attention, comprehensive data on the prevalence, resistance gene distribution, and co-occurrence patterns of ESBL-producing and colistin-resistant *E. coli* in retail meat remain scarce in Türkiye. Addressing this gap is essential to better understand the role of the food chain in the dissemination of resistance and to inform effective surveillance and control strategies.

Resistance to extended-spectrum cephalosporins (ESCs) in Enterobacterales, mostly mediated by ESBLs or plasmidic *AmpC* (pAmpC), is a major problem in both human and veterinary medicine. Since the late 1990s, ESBL-producing *E. coli* (ESBL-EC) has been detected in retail meat and production animals across Europe, Asia, Africa, and the United States [2,3]. The World Health Organization (WHO) has emphasized that third-generation cephalosporin-resistant *Enterobacteriaceae*, including those producing ESBLs, represent one of the most critical challenges of the 21st century. WHO has recommended integrating global surveillance of ESBL-producing *E. coli* through a “One Health” approach, addressing both human and animal health aspects while highlighting implementation strategies and potential interventions [4].

The most significant class A enzymes in clinical settings are known as ESBLs. The three primary families identified are TEM, SHV, and CTX-M types [5]. TEM and SHV-type ESBLs emerge from the original narrow-spectrum TEM-1/-2 or SHV-1 beta-lactamases through amino acid substitutions, whereas all CTX-M enzymes inherently display an ESBL phenotype [6]. ESBL-producing *E. coli* strains exhibit resistance to third-generation cephalosporins such as ceftriaxone, ceftazidime, and ceftiofur, as well as fourth-generation cephalosporins like cefepime, cefpirome, and cefquinome, and also to aztreonam. Until the 1990s, SHV and TEM types were the predominant ESBLs identified in human populations. However, in more recent years, CTX-M enzymes, especially CTX-M-15, have emerged as the most common ESBL variants [7].

The most common ESBL genotypes in food-producing animals are *blaCTX*-M, followed by *blaSHV*-12 and *blaTEM*-52 [8]. Various ESBL genotypes, including *blaTEM*-1, *blaTEM*-52, *blaSHV*-2, *blaSHV*-5, *blaSHV*-12, *blaCTX-M*-1, *blaCTXM*-2, *blaCTX-M*-3, *blaCT-M*-8, *blaCTXM*-14, *blaCTXM*-15, *blaCTX-M*-55, and *blaCTX-M*-123, have been reported in chicken, turkey, beef, and pork in several countries, including China, Japan, Tunisia, and the UK [3,9,10,11,12].

Colistin, which belongs to the polymyxin group of polypeptide antibiotics, is of great public health importance as it is considered a last-resort antimicrobial agent for Gram-negative bacteria. Since the discovery of the plasmid-mediated *mcr*-1 gene in human and animal isolates in China in 2015, several studies have reported an increasing trend in the coexistence of *mcr* genes and ESBL/AmpC genes in the same bacterial isolates [13]. Globally, mcr-mediated colistin resistance is more frequently reported in food-producing animals, particularly poultry, than in humans. This indicates that livestock serve as significant reservoirs for many critically important resistant strains [14]. Moreover, *mcr*-1-positive and/or ESBL/pAmpC-producing *E. coli* strains from healthy broilers were previously considered commensals. However, recent studies have shown that some of these strains also carry virulence-associated genes characteristic of avian pathogenic *E. coli* (APEC) or extraintestinal pathogenic *E. coli* (ExPEC)-like strains.

To our knowledge, this is the first study in Türkiye to investigate the co-occurrence of extended-spectrum β-lactamase (ESBL) production and colistin resistance both phenotypically and genotypically in *E. coli* from diverse retail meat sources. Accordingly, this study aimed to characterize resistance profiles and to determine the distribution of key genes (*blaCTX-M*, *blaOXA*, *blaTEM*, *blaSHV*, *mcr*-1, *mcr*-2, *mcr*-3) in order to assess the potential public health risks posed by resistant *E. coli* in the food supply.

## 2. Materials and Methods

### 2.1. Sample Collection

In this study, samples distributed throughout Turkiye and available for consumption in markets, supermarkets, and butcher shops of various sizes were used. A total of 180 retail samples were purchased, including chicken (*n* = 60; parts, n = 40; internals, *n* = 10; processed products, *n* = 10), lamb (n = 19; minced meat, n = 8; meat cuts, *n* = 2; internals, n = 9), beef (*n* = 41; minced meat, *n* = 19; meat cuts, *n* = 13; internals, *n* = 9), and fish (*n* = 60). All samples were transported to the laboratory in refrigerated containers with ice packs and processed within a maximum of 2 h. Sample size adequacy was evaluated using G*Power 3.1, assuming a medium effect size (Cohen’s f = 0.25), significance level of 0.05, and 80% power, which indicated a minimum of 52 samples per group. The selection of f = 0.25 was based on conventional benchmarks for medium effect sizes frequently applied in microbiological and food safety studies [15,16]. Accordingly, 60 samples per group were included to ensure reliable comparisons.

### 2.2. Isolation of ESBL E. coli

The isolation procedure was performed as described by Ongut et al. [17] with slight modifications. Accordingly, 25 g of each sample was weighed into a sterile, filtered stomacher bag and homogenized with 225 mL of Buffered Peptone Water (Neogen, NCM0015A, Lansing, MI, USA) in the stomacher for 90 s. After incubating the homogenate in BPW at 37 °C for 18–24 h, 100 µL of the homogenate was plated on ESBL Chromogenic Agar (Condolab, Madrid, Spain) and Petri dishes were incubated at 37 °C for 18–24 h. Suspicious colonies that grew pink on the chromogenic medium were selected and cultured on blood agar and then on Tyriptic Soy Agar (TSA, Merck, Darmstadt, Germany) by the smear plate method. DNA extraction was performed from the colonies grown on TSA.

### 2.3. Phenotypic Confirmation of ESBL

To phenotypically confirm ESBL production, combined disk tests were performed using cefotaxime (5 µg, Oxoid, Hampshire, UK) and ceftazidime (10 µg, Oxoid, UK) alone and in combination with clavulanic acid, following standard protocols. The confirmation was further supported using a commercial combination disk kit (Oxoid, UK), which contained cefpodoxime (10 µg) alone and in combination with clavulanic acid (10 µg). An increase of ≥5 mm in the inhibition zone diameter for the cefpodoxime–clavulanic acid disk compared with the cefpodoxime disk alone was interpreted as indicative of ESBL production, in accordance with the manufacturer’s guidelines and CLSI recommendations [18]. From each positive sample, five presumptive ESBL-producing colonies were selected for further analysis. These colonies were subcultured on Plate Count Agar (PCA, Merck, Germany), and preserved by suspension in Brain Heart Infusion (BHI, Oxoid, UK) broth enriched with 15% glycerol. Stocks were stored at −20 °C for subsequent molecular analysis. *E. coli* ATCC^®^ 25922 (American Type Culture Collection, Manassas, VA, USA) was used as the quality control strain. Quality control procedures were performed at the start of each batch of susceptibility testing and whenever a new lot of media or antibiotic disks was introduced. Zone diameters for QC strains were required to fall within the CLSI and EUCAST recommended ranges, and inter-batch consistency was ensured by confirming that repeated QC results remained within these acceptance limits.

### 2.4. DNA Extraction and Molecular Characterization

The boiling method was used for DNA extraction from the isolates. *E. coli* species-specific *uspA* gene primers published by Chen and Griffiths [19] were used for species-level confirmation of suspected *E. coli* isolates. The primer pairs and lengths of the β-lactamase and colistin resistance genes are presented in Table 1.

Multiplex PCR thermal cycler temperature cycling of β-lactamase genes was performed at 95 °C for 15 min for initial denaturation, followed by 30 cycles of 94 °C for 30 s, 62 °C for 90 s, and 72 °C for 60 s, with a final extension at 72 °C for 10 min. The amplified PCR products were run on a 1% agarose gel in an electrophoresis tank for visualization. PCR cycling was performed as described in [19,20,21,22,23,24,25,26,27,28]. PCR products were analyzed by electrophoresis on 1% agarose gels. For isolates positive for *blaCTX*-*M*, further subtyping was performed using group-specific primers for *blaCTX-M*1, *blaCTX-M*2, *blaCTX-M*9, and *blaCTX-M*8/25.

### 2.5. Determination of Colistin Resistance

Antimicrobial susceptibility of all isolates was assessed using broth microdilution in accordance with the guidelines set by the European Committee on Antimicrobial Susceptibility Testing (EUCAST). Minimal inhibitory concentrations (MICs) of the isolates were determined using broth microdilution in 96-well plates. Colistin (ComASP, Liofilchem, Roseto degli Abruzzi, Italy). The bacterial inocula were prepared on Muller-Hinton Agar, inoculated in MHB tubes, and adjusted to 0.5 McFarland standard. The microdilution plates were incubated for 20–24 h at 37 °C. *E. coli* NCTC^®^ 13846 (National Collection of Type Cultures, Public Health England, London, UK) strain was used as a reference strain. Quality control testing was performed at the start of each new testing batch and whenever new lots of media or reagents were introduced. MIC values for QC strains were required to fall within the EUCAST-defined acceptance ranges, and inter-batch reproducibility was verified to ensure consistent results across assays. The resistance cut-off value for colistin (>2 μg/mL) was evaluated according to EUCAST [29].

### 2.6. Detection of Antimicrobial Resistance Profiles

The susceptibility of ESBL-producing *E. coli* isolates to selected β-lactam group antibiotics (penicillins, monobactams, cephalosporins, carbapenems) was determined by the Kirby–Bauer agar disk diffusion method on Mueller-Hinton agar. The panel included Ampicillin (aminopenicillins), Amoxicillin–clavulanic acid (aminopenicillins), Aztreonam (monobactams), Cefepime (4th generation cephalosporins), Moxalactam (3rd generation cephalosporins), Cefpodoxime (3rd generation cephalosporins), Cefuroxime (2nd generation cephalosporins), Cephalothin (1st generation cephalosporins), Imipenem (carbapenems), and Meropenem (carbapenems). Zone diameters were interpreted according to the Clinical and Laboratory Standards Institute [18] breakpoints for all β-lactam agents. The EUCAST guidelines [29] were applied for colistin, with isolates classified as resistant when MIC values were >2 µg/mL. *E. coli* ATCC^®^ 25922 was used as the quality control strain, tested with each new batch of media and antibiotic disks to ensure performance compliance with reference standards.

### 2.7. Statistical Analysis

Statistical analyses were conducted to evaluate the association between sample types and the distribution of resistance genes, as well as to explore potential co-occurrence patterns among these resistance genes. Given the presence of small sample sizes and expected cell counts < 5 in several categories, Fisher’s exact test was chosen as the primary method for all categorical comparisons (e.g., presence of *mcr*-1 and sample type, co-occurrence of *blaCTX*-*M*1 and *blaTEM*). Pairwise associations among resistance genes were further tested using Fisher’s exact test (*p* < 0.05) with the Benjamini–Hochberg correction to control the false discovery rate, and significant interactions were visualized in a co-occurrence network using (v3.10.12) and NetworkX (v3.2.1). The Multiple Antibiotic Resistance (MAR) index was calculated for each isolate as the ratio of the number of antibiotics to which the isolate was resistant to the total number of antibiotics tested (*n* = 16). Mean MAR values were then computed per sample type. Statistical significance was set at *p* < 0.05. All analyses were performed using IBM SPSS Statistics v26.0 (IBM Corp., Armonk, NY, USA).

## 3. Results

In total, 61 out of 180 samples (33.9%) yielded presumptive ESBL-producing isolates. Among the 61 presumptive isolates, 39 (21.6%) were confirmed as *E. coli* by *usp*A gene amplification, while the remaining 22 were negative for *E. coli* confirmation and thus excluded from subsequent molecular analyses. The distribution varied among sample types: chicken internals (60%, 6/10) and chicken parts (65%, 26/40) showed the highest prevalence, followed by lamb minced meat (12.5%, 1/8), and beef internals (22.2%, 2/9). Lower rates were observed in beef minced meat (5.26%, 1/19) and lamb internals (11.1%, 1/9), while fish displayed the lowest positivity (3.3%, 2/60). No ESBL-producing isolates were detected in chicken processed products (0/10), lamb meat (0/2), and beef meat (0/13) (Supplementary Table S1).

All confirmed ESBL *E. coli* carried *blaCTX-M* (100%), exclusively belonging to the *blaCTX-M-1* group. In addition, *blaTEM* was identified in 24 isolates (61.5%), and blaOXA was identified in 2 isolates (5.1%). Within the chicken internals (CI), ESBL and *usp*A positive isolates originated from liver (*n* = 3), heart (*n* = 2), and gizzard (*n* = 1), while in chicken parts (CP), confirmed isolates were derived from thigh (*n* = 12), drumstick (*n* = 1), wing (*n* = 8), breast (*n* = 4), and neck (*n* = 1). All of these isolates carried *blaCTX-M* and *blaCTX-M*1. Distribution of *blaTEM*-positive isolates showed product-specific variation, being detected in chicken liver (*n* = 1), wing (*n* = 5), breast (*n* = 2), thigh (*n* = 10), and drumstick (*n* = 1). For *mcr*-1, three positive isolates were identified among chicken parts, specifically from the wing (*n* = 2) and drumstick (*n* = 1).

Colistin resistance was phenotypically detected in 6 isolates (15.4%), originating from chicken parts (*n* = 3) and lamb minced meat (*n* = 3). Five isolates exhibited MIC values of 4 µg/mL, and one isolate showed 16 µg/mL, corresponding to an MIC_50_ of 4 µg/mL and an MIC_90_ of 16 µg/mL according to EUCAST [28] breakpoints (>2 µg/mL). All resistant isolates carried *mcr*-1, while one lamb minced meat isolate additionally harbored *mcr*-2 (2.6%).

**Figure 1:**
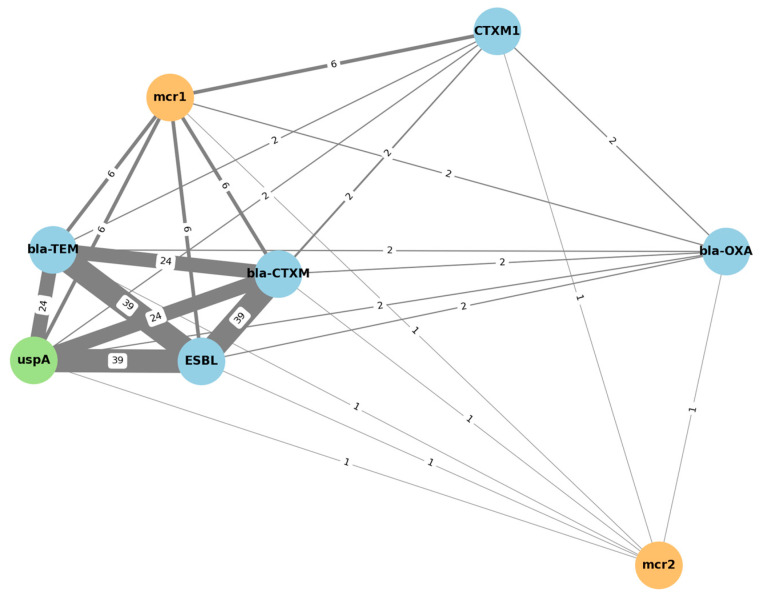
Clustered co-occurrence network of resistance genes. Blue: ESBL-related genes; orange: colistin resistance genes; green: *E. coli* marker gene. The numbers displayed along the connecting lines indicate the number of isolates in which both genes co-occurred. Thicker lines represent stronger associations between resistance determinants.

A co-occurrence network analysis (Figure 1.) was performed using presence/absence matrices of resistance genes across isolates. Edges were drawn between genes that co-occurred in at least two isolates. The network was generated with Python (NetworkX library) and visualized with a force-directed layout (spring algorithm). Node sizes were proportional to gene prevalence, and edge weights reflected co-occurrence frequency. Statistical significance of associations was evaluated with Fisher’s exact test (*p* < 0.05, Benjamini–Hochberg correction for multiple testing).

The *bla-OXA* gene exhibited low co-occurrence rates (*n* = 2) and remained topologically peripheral in the network, while *mcr-*2 was rarely detected and did not significantly co-cluster with any other gene (*n* = 1). This network visualization supports the hypothesis that ESBL production and colistin resistance may coexist within certain isolates, particularly those harboring *mcr-*1, but not *mcr-*2.

**Figure 2:**
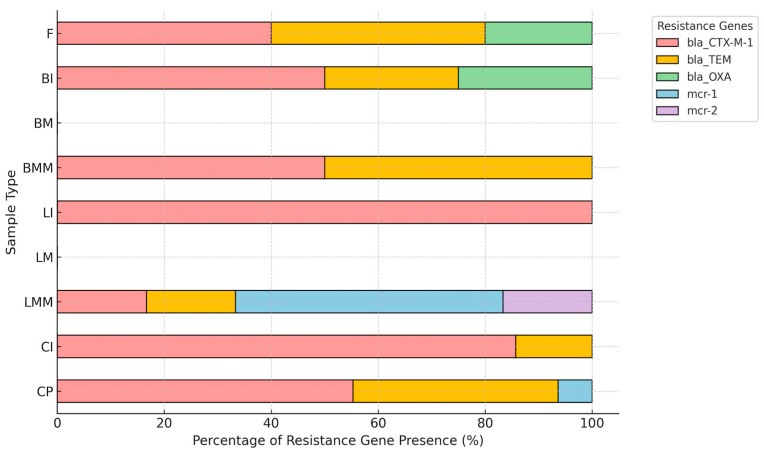
Co-occurrence patterns of resistance genes in ESBL *E. Coli* by sample type. CP (chicken parts), CI (chicken internals), CPR (chicken processed products), LMM (lamb minced meat), LM (lamb meat), LI (lamb internals), BMM (beef minced meat), BM (beef meat), BI (beef internals), F (fish). Distribution of resistance genes in ESBL-producing *E. coli* isolates by sample type, expressed as relative percentages within each category.

Co-occurrence analysis of resistance genes revealed distinct patterns among sample types (Figure 2). The combination of *blaCTX-M* and *blaCTX-M-*1 was most frequently detected in poultry-derived isolates (CP, CI). A co-occurrence of *blaCTX-M-*1 with *mcr-*1 was observed in lamb minced meat (LMM). In addition, co-occurrence of *blaTEM* and *mcr-*1 was detected in a limited number of isolates.

**Figure 3:**
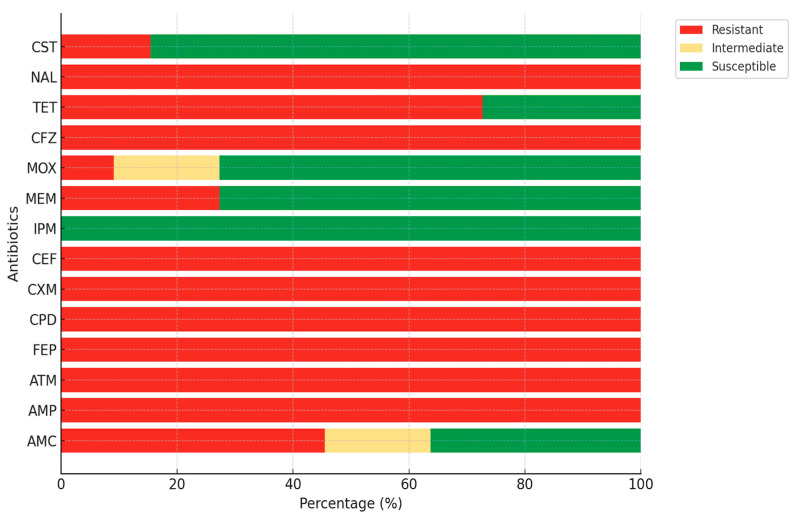
Antibiotic resistance profiles of ESBL *E. coli* isolates. AMP, ampicillin; AMC, amoxicillin-clavulanic acid; ATM, aztreonam; CPD, cefpodoxime; CXM, cefuroxime; CFZ, cephalothin; FEP, cefepime; CEF, cephazolin; MOX, moxalactam; MEM, meropenem; IPM, imipenem; TET, tetracycline; NAL, nalidixic acid; CST, colistin.

The antimicrobial susceptibility patterns of isolates demonstrated exceptionally high resistance to β-lactam antibiotics. All isolates (100%) were resistant to Ampicillin, Aztreonam, Cefepime, Cefpodoxime, Cefuroxime, Cephalothin, and Cephazolin. Resistance to Amoxicillin-Clavulanic Acid was 45.5%, while 18.2% of isolates showed intermediate susceptibility. Although Imipenem retained full efficacy (100% susceptible), Meropenem resistance was observed in 27.3% of the isolates. Resistance to Moxalactam was lower (9.1%), with 72.7% of isolates remaining susceptible. Among non-β-lactam agents, resistance to Tetracycline (72.7%) and Nalidixic Acid (100%) was strikingly high (Figure 3).

Among the 39 ESBL-producing *E. coli* isolates, colistin resistance was identified in six (15.4%) based on MIC values determined via broth microdilution. Five isolates exhibited MIC values of 4 µg/mL, and one isolate showed 16 µg/mL, resulting in an MIC_50_ of 4 µg/mL and an MIC_90_ of 16 µg/mL. All values exceeded the established clinical breakpoint for *E. coli*. According to EUCAST guidelines [29], isolates with MIC values > 2 µg/mL are classified as resistant to colistin.

**Figure 4:**
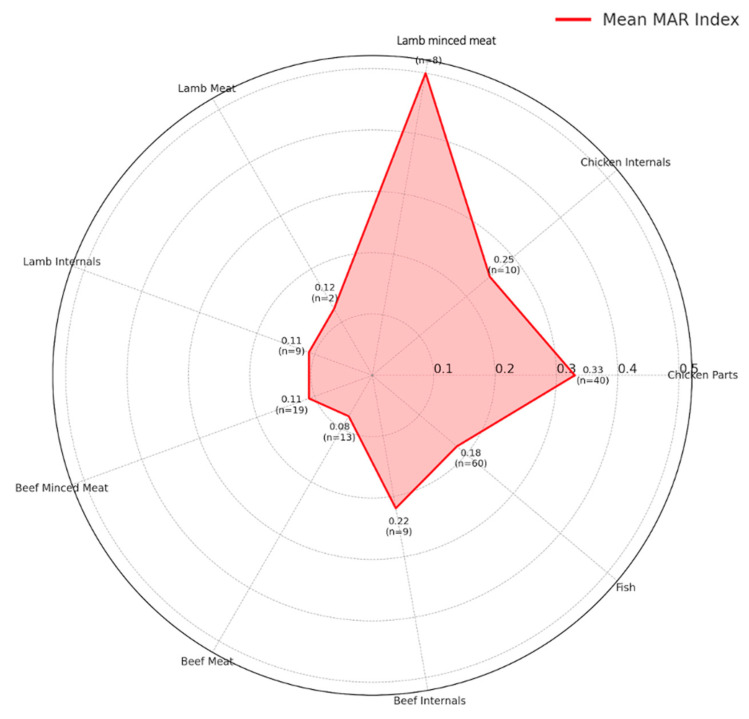
Radar plot of mean MAR index per sample type.

The radar plot (Figure 4) illustrates the mean MAR (Multiple Antibiotic Resistance) index for *E. coli* isolates obtained from various retail meat types. Each axis represents a different sample type (e.g., chicken parts, beef meat, fish, etc.). The red line shows the average MAR index for that category, while the values are also annotated numerically along with the number of isolates (n) contributing to the mean. The MAR index was calculated as the ratio of the number of antibiotics to which an isolate was resistant to the total number of antibiotics tested (*n* = 16 in this study). For example, an isolate resistant to 8 out of 16 antibiotics was assigned a MAR index of 0.50. This representation highlights that lamb minced meat (MAR: 0.50) and chicken parts (MAR: 0.33) harbor the highest resistance burdens, while beef meat and internals exhibit comparatively lower MAR scores.

## 4. Discussion

This study demonstrated a substantial prevalence (21.6%) of ESBL-producing *E. coli* in retail meat, with poultry emerging as the dominant reservoir. The highest detection rates in chicken parts (65%) and giblets (60%) highlight poultry, particularly internal organs, as major carriers of resistant bacteria. These findings align with European reports, where cefotaxime-resistant *E. coli* was most frequently detected in chicken and turkey meat (74.9% and 40.1%, respectively) compared to beef, pork, and minced meat (4.2–15.3%) in Germany [30], and ESBL-producing *E. coli* occurred in 91.7% of chicken samples in France [31]. Lower prevalence of non-poultry meats (22.2% in beef internals (2/9), 5.26% in beef minced meat (1/19), 11.1% in lamb internals (1/9), and only 3.3% in fish (2/60)) was consistent with prior reports indicating poultry’s dominant role in ESBL dissemination [11,32]. Evidence from other regions further supports this trend: in Italy, Musa et al. [33] reported ESBL-producing *E. coli* in 18.6% of chicken isolates, the highest in conventional flocks; in Lithuania, Klimienė et al. [34] found *E. coli* in 92.7% of poultry meat samples, more than half being ESBL producers; and in India, Hussain et al. [35] detected ESBL-producing *E. coli* in 46% of broiler meat but only 15% of free-range meat, with multidrug resistance far more frequent in broilers. Collectively, these findings confirm that poultry consistently carries the highest burden of ESBL-producing *E. coli*, whereas non-poultry meats play a comparatively minor role in the dissemination of resistance along the food chain.

The particularly low rate in fish likely reflects fundamental differences between aquaculture and terrestrial animal farming. In aquaculture, antimicrobial use is often more restricted, production cycles are shorter, and environmental dilution effects, such as water exchange, may reduce bacterial loads. By contrast, terrestrial livestock production involves more frequent and prolonged antimicrobial exposure, higher stocking densities, and greater opportunities for fecal contamination, all of which can promote the emergence and persistence of resistant strains [36].

Molecular characterization confirmed the absolute predominance of *blaCTX-M* genes, exclusively *blaCTX-M-1*, among *E. coli* isolates. This reflects the global trend of *blaCTX-M* supplanting *blaTEM* and *blaSHV* variants in foodborne *E. coli*. The predominance of *blaCTX-M* in poultry-associated ESBL-producing *E. coli* is attributed to their frequent carriage on highly transferable plasmids such as IncI1-ST3, which facilitate efficient horizontal gene transfer [37,38]. The use of third-generation cephalosporins, such as ceftiofur, in veterinary therapy exerts strong selective pressure, favoring the survival and expansion of CTX-M-producing strains [39,40].

The detection of *blaTEM* (61.5%) indicates a substantial but secondary role compared to *blaCTX-M*, while *blaOXA* was rarely observed (5.1%), consistent with its limited mobility in food reservoirs. *blaTEM* was identified in 61.5% (24/39) of tested isolates, while *blaOXA* was found in only 5.13% (2/39) of tested isolates. This supports global trends indicating a shift from earlier ESBL types, such as *blaSHV* and *blaTEM*, toward *blaCTX-M* type enzymes, which now dominate as the primary resistance mechanism in both clinical and foodborne *E. coli* isolates [3,7,37]. The absence of *blaSHV* and the low detection rate of *blaOXA* are consistent with current data, which suggest that these genes are less commonly found in foodborne *E. coli* isolates, particularly compared to the widespread prevalence of *blaCTX-M* variants [22,23].

The less frequent detection of other ESBL types, *blaSHV*, *blaTEM*, and *blaOXA,* is likely due to their lower mobility and limited adaptive advantage under current antimicrobial use patterns [41,42]. Nevertheless, regional variation exists. In Colombia, Martins et al. [43] reported *blaCTX-M* and *blaTEM* co-carriage in 78.9% of minced meat isolates, while in South Korea, Lim et al. [44] observed that all ESBL-producing *E. coli* from chicken carcasses harbored *blaTEM*, but only 40.3% carried *blaCTX-M* group 1 genes. Similarly, in a German retail poultry study, Kola et al. [45] found *blaSHV*-12 (*n* = 82) slightly exceeding *blaCTX-M*1 (*n* = 77). These examples illustrate that although *blaCTX-M*1 dominates globally, other ESBL variants such as *blaTEM* and *blaSHV* can emerge as regionally dominant depending on ecological, production, or food matrix conditions.

Antimicrobial susceptibility testing revealed a consistent resistance profile across all isolates. All isolates exhibited resistance to ampicillin and third-generation cephalosporins, including cefpodoxime and moxalactam. More strikingly, every isolate was resistant to cefepime, a fourth-generation cephalosporin widely used in clinical settings. This trend narrows effective treatment options and amplifies the clinical implications of foodborne ESBL-producing *E. coli*. Carbapenems (imipenem and meropenem) remained fully susceptible, with no resistant isolates detected, emphasizing their continued efficacy as last resort agents. Similar resistance patterns have been reported globally. For example, Martins et al. [43] observed that nearly all minced meat isolates (94.7%) in their study were resistant to cefotaxime, while all remained susceptible to imipenem and colistin, mirroring our findings on carbapenem susceptibility. Likewise, Casella et al. [31] documented widespread co-resistance among ESBL-producing *E. coli* from French chicken meat, particularly to sulfonamides (84.4%), tetracyclines (75.3%), and trimethoprim (51.9%), highlighting a similar multi-drug resistance profile extending beyond β-lactams. Moreover, Guo et al. [46] identified aminoglycoside resistance genes in 92.4%, sulfonamide resistance genes in 86.2%, and colistin resistance genes (e.g., *mcr-*1, *mcr-*3, and *mcr-*5) in 15.6% of isolates from various meat sources in Singapore, suggesting comparable co-selection of multiple resistance determinants across regions. Similarly, Liu et al. [26] reported that among 408 ESBL-producing *E. coli* isolates from retail meats in China, 70.3% were resistant to ciprofloxacin, 66.2% to tetracycline, and 52.5% to chloramphenicol, while all remained susceptible to meropenem, consistent with the pattern observed in our study.

Detection of colistin resistance (15.4%, 6/39) revealed *mcr-*1 carriage in all resistant isolates, predominantly from chicken meat, while one lamb minced meat isolate additionally harbored *mcr-*2, representing a rare co-detection pattern. Poultry remains a well-documented reservoir of plasmid-mediated colistin resistance [26,47], whereas the simultaneous detection of *mcr-*1 and *mcr-*2 in lamb meat is unusual and broadens the known host range of these genes. This finding is particularly remarkable, as *mcr-*2 has predominantly been reported in pigs and only sporadically in cattle [48]. Its occurrence in lamb minced meat therefore suggests atypical transmission dynamics, potentially linked to cross-contamination during processing, environmental exposure, or plasmid-mediated horizontal gene transfer between different livestock species. Collectively, these results highlight the ongoing public health risk posed by *mcr* genes in retail meat, especially when located on multidrug-resistant plasmids capable of horizontal dissemination even in the absence of direct colistin use [49].

In terms of gene co-occurrence, distinct patterns were observed among sample types. *blaCTX-M-*1, the sole CTX-M lineage identified, was most prevalent in poultry-derived isolates, consistent with its recognized dominance in chicken meat. Notably, *blaCTX-M-*1 co-occurred with *mcr-*1 exclusively in lamb minced meat, implying that small ruminant products may act as occasional reservoirs of multidrug resistance. The rare co-localization of *blaTEM* and *mcr-*1 further supports sporadic overlap between β-lactamase and colistin resistance determinants in foodborne isolates.

A recurring pattern in the data was the co-occurrence of *mcr*-1 and *blaCTX-M*1 within the same isolates, illustrating the convergence of resistance to critically important β-lactams and polymyxins, a pattern increasingly reported across different countries and production systems. Such a linkage raises concerns about the stable maintenance of multidrug resistance within the food chain. Importantly, the co-occurrence of *blaCTX-M*1 and *mcr-*1 in all colistin-resistant isolates demonstrates a convergence of resistance to critically important antimicrobials, raising concerns about the stability of multidrug resistance in foodborne reservoirs. Although *mcr-*2 was detected in one isolate, its presence even at low frequency may reflect an expanding diversity of colistin resistance mechanisms in the food chain [13]. The coexistence of *mcr*-1 and *bla*CTX-*M*1 in all colistin-resistant isolates underscores the convergence of resistance to both last-resort antibiotics, polymyxins, and extended-spectrum β-lactams. This pattern may enhance persistence and dissemination under diverse selective pressures. A similar pattern was also reported by Madni et al. [49], who found that 88% of *E. coli* isolates from commercial broiler chickens in Pakistan harbored *mcr*-1, while 77% carried *blaCTX*-M, with a substantial number (33/38). Similarly, Songsaeng et al. [50] identified co-occurrence of *mcr*-1, *mcr*-3, and *blaCTX*-M in 94% of *E. coli* isolates from pig farms, suggesting a frequent genetic linkage and potential horizontal transmission. In another investigation, colistin-resistant *E. coli* isolated from chicken meat in Bangladesh were found to co-carry *mcr*-1 and *blaTEM*, suggesting the concurrent presence of resistance to both polymyxins and β-lactams [51]. Taken together, these findings are consistent with our own results, where all colistin-resistant isolates were *blaCTX-M*1 positive, and half additionally harbored *blaTEM*, indicating a complex and potentially synergistic resistance profile. The frequent co-detection of ESBL and *mcr* genes across geographically and agriculturally diverse settings reflects the widespread nature of co-resistance and its occurrence in multiple reservoirs. This pattern has been associated with the maintenance of these genes on mobile genetic elements, which can facilitate the persistence and spread of multidrug resistance even in the absence of direct antibiotic pressure. In line with this observation, Zhao et al. [52] demonstrated that *mcr*-1 and *blaCTX-M*55 could be co-transferred via conjugative plasmids in *E. coli* isolates, and highlighted the role of insertion sequences such as IS26 and ISEcp1 in mediating plasmid fusion, enabling resistance gene dissemination independent of ongoing selective pressure. Taken together, these findings confirm poultry as the principal reservoir of ESBL-producing *E. coli* in retail meat, while also revealing the unexpected detection of resistant isolates in lamb minced meat. This unexpected occurrence may be explained by several factors, including cross-contamination during slaughter or processing, horizontal transfer of resistance plasmids across livestock species, or underrecognized antimicrobial use practices in small ruminant production. The exclusive detection of *blaCTXM*-1, combined with sporadic but alarming co-occurrence of *mcr* genes, underlines the need for integrated monitoring across different meat types and for strict enforcement of antimicrobial stewardship in animal production.

The MAR index analysis further supports the molecular and phenotypic findings by revealing that poultry and lamb minced meat exhibited the highest levels of multidrug resistance, consistent with their higher prevalence of ESBL and *mcr* genes. Elevated MAR scores in these products indicate cumulative exposure to multiple antimicrobial classes, likely reflecting selective pressures within intensive production systems and cross-contamination during processing. In contrast, the comparatively low MAR indices in beef and fish samples align with their lower detection rates of β-lactamase and colistin resistance genes, underscoring the species-specific variability in antimicrobial resistance within the retail meat supply chain.

## 5. Conclusions

The detection of ESBL and plasmid-mediated colistin resistance genes in *E. coli* from retail meat demonstrates the convergence of resistance to critically important antibiotics. Poultry, particularly internal organs, were the main reservoir, but the presence of *mcr*-positive isolates in lamb minced meat highlights that resistance determinants can also appear in less expected sources. The predominance of *blaCTX-M*1 and its frequent co-detection with *mcr*-1 suggest a role of mobile genetic elements in sustaining multidrug resistance.

These conclusions should be considered in light of certain limitations, including the cross-sectional design, potential sampling bias, and the absence of whole-genome sequencing, which restrict generalizability and the depth of genetic characterization. Overall, the study underscores an evolving resistance landscape in foodborne pathogens and emphasizes the importance of continued One Health-based surveillance while acknowledging these methodological constraints.

## Figures and Tables

**Table 1:** Primer pairs used in the study.

Gene Region	Primer Sequence (5′-3′)	Length (bp)	Reference
*usp*A	5′CCGATACGCTGCCAATCAGT3′ 5′ACGCAGACCGTAGGCCAGAT3′	884	[19]
ESBL			
*bla_SHV_*	CTTTATCGGCCCTCACTCAA AGGTGCTCATCATGGGAAAG	237	[20]
*bla_TEM_*	CGCCGCATACACTATTCTCAGAATGA ACGCTCACCGGCTCCAGATTTAT	445	[21]
*bla_OXA_*	ACACAATACATATCAACTTCGC AGTGTGTTTAGAATGGTGATC	813	[22]
*bla_CTX-M_*	ATGTGCAGYACCAGTAARGTKATGGC TGGGTRAARTARGTSACCAGAAYCAGCGG	593	[23]
*bla_CTX_*_-*M*1_	CGTCACGCTGTTGTTAGGAA TCGGTTCGCTTTCACTTTTC	227	[20]
*bla_CTX_*_-*M*2_	GGAGAAAAGTTCGGGAGGTC GCTTATCGCTCTCGCTCTGT	155	[24]
*bla_CTX_*_-*M*9_	ACGTGGCTCAAAGGCAATACCGGCTG GGTAAAATAGGTCA	174	[24]
*bla_CTX_*_-*M*8/25_	AACRCRCAGACGCTCTAC TCGAGCCGGAASGTGTYAT	326	[25]
MCR			
*mcr-*1	CGGTCAGTCCGTTTGTTC CTTGGTCGGTCTGTAGGG	309	[26]
*mcr-*2	TGTTGCTTGTGCCGATTGGA AGATGGTATTGTTGGTTGCTG	567	[27]
*mcr-*3	AAATAAAAATTGTTCCGCTTAT GAATGGAGATCCCCGTTTTT	930	[28]

## Data Availability

The original contributions presented in the study are included in the article/supplementary material, further inquiries can be directed to the corresponding author.

## Supplementary Materials

The following supporting information can be downloaded at: https://www.mdpi.com/article/10.3390/foods14203573/s1.
