# Supplementary material for: Co-Occurrence and Molecular Characterization of ESBL-Producing and Colistin-Resistant Escherichia coli Isolates from Retail Raw Meat
Source: Foods. 2025 Oct 21;14(20):3573. doi: 10.3390/foods14203573 (PMC12564342; doi:10.3390/foods14203573)
Supplement: Supplementary file 1 [file foods-14-03573-s001.zip › foods-3910269-supplementary.pdf]

Supplementary Table S1. Distribution of ESBL positive *E. coli* isolates and antimicrobial resistance genes.

**Samples**	**ESBL**	***uspA***	***bla-CTXM***	***CTXM1***	***bla-TEM***	***bla-OXA***	***mcr1***	***mcr2***
CP (n=40)	30	26	26	26	18	nd	3	nd
---Thigh (n=x)		*12*	*12*	*12*				
---Drumstick (n=x)		*1*	*1*	*1*			*1*	
---Wing (n=x)		*8*	*8*	*8*			*2*	
---Breast (n=x)		*4*	*4*	*4*				
---Neck (n=x)		*1*	*1*	*1*				
CI (n=10)	10	6	6	6	1	nd	nd	nd
---Heart (n=x)		*2*	*2*	*2*				
---Liver (n=x)		*3*	*3*	*3*				
---Gizzard (n=x)		*1*	*1*	*1*				
CPR (n=10)	nd	nd	nd	nd	nd	nd	nd	nd
LMM (n=8)	5	1	1	1	1	nd	3	1
LM (n=2)	1	nd	nd	nd	nd	nd	nd	nd
LI (n=9)	2	1	1	1	nd	nd	nd	nd
BMM (n=19)	5	1	1	1	1	nd	nd	nd
BM (n=13)	2	nd	nd	nd	nd	nd	nd	nd
BI (n=9)	4	2	2	2	1	1	nd	nd
F (n=60)	2	2	2	2	2	1	nd	nd
**Total**	**61**	**39**	**39**	**39**	**24**	**2**	**6**	**1**

ND*: Not Detected; CP: Chicken Parts; CG: Chicken Internals; CPR: Chicken Products; LMM: Lamb Minced Meats; LM: Lamb Meat; LI: Lamb Internals; BMM: Beef Minced Meat; BM:Beaf Meat; BI: Beef Internals; F: Fish.
